# Influence of muscle fiber type composition on early fat accumulation under high-fat diet challenge

**DOI:** 10.1371/journal.pone.0182430

**Published:** 2017-08-01

**Authors:** Ning Hua, Hirokazu Takahashi, Grace M. Yee, Yoichiro Kitajima, Sayaka Katagiri, Motoyasu Kojima, Keizo Anzai, Yuichiro Eguchi, James A. Hamilton

**Affiliations:** 1 Department of Physiology and Biophysics, Boston University School of Medicine, Boston, MA, United States of America; 2 Research Division, Joslin Diabetes Center and Department of Medicine, Harvard Medical School, Boston, Massachusetts, United States of America; 3 Division of Internal Medicine, Saga Medical School, Saga, Japan; 4 Clinical Gastroenterology, Eguchi Hospital, Saga, Japan; 5 Division of Hepatology, Saga Medical School, Liver Center, Saga, Japan; 6 Department of Biomedical Engineering, Boston University, Boston, Massachusetts, United States of America; University of Minnesota Medical Center, UNITED STATES

## Abstract

**Objective:**

To investigate whether differences in muscle fiber types affect early-stage fat accumulation, under high fat diet challenge in mice.

**Methods:**

Twelve healthy male C57BL/6 mice experienced with short-term (6 weeks) diet treatment for the evaluation of early pattern changes in muscular fat. The mice were randomly divided into two groups: high fat diet (n = 8) and normal control diet (n = 4). Extra- and intra-myocellular lipid (EMCL and IMCL) in lumbar muscles (type I fiber predominant) and tibialis anterior (TA) muscle (type II fiber predominant) were determined using magnetic resonance spectroscopy (MRS). Correlation of EMCL, IMCL and their ratio between TA and lumbar muscles was evaluated.

**Results:**

EMCL increased greatly in both muscle types after high fat diet. IMCL in TA and lumbar muscles increased to a much lower extent, with a slightly greater increase in TA muscles. EMCLs in the 2 muscles were positively correlated (r = 0.84, p = 0.01), but IMCLs showed a negative relationship (r = -0.84, p = 0.01). In lumbar muscles, high fat diet significantly decreased type I fiber while it increased type II fiber (all p≤0.001). In TA muscle, there was no significant fiber type shifting (p>0.05).

**Conclusions:**

Under short-time high fat diet challenge, lipid tends to initially accumulate extra-cellularly. In addition, compared to type II dominant muscle, Type I dominant muscle was less susceptible to IMCL accumulation but more to fiber type shifting. These phenomena might reflect compensative responses of skeletal muscle to dietary lipid overload in order to regulate metabolic homeostasis.

## Introduction

Obesity is characterized by fat accumulation in many sites such as the liver, heart and skeletal muscles [1]. It is well studied that regional fat accumulation within skeletal muscle correlates with insulin resistance independently of visceral fat accumulation and total body fat mass in humans [2,3]. Therefore, exploring patterns of regional fat accumulation may help better stratify obesity into sub-types and predict their corresponding metabolic consequences. The buildup of skeletal muscle adiposity is known to be linked to excess dietary lipids[6], but little is understood about the impact and complications from fiber type composition, which is characterized by its myosin heavy chain isoforms [7]. Type I fibers twitch slowly and are predominantly red tonic muscles. Type II fibers are predominantly white muscles and optimized for fast movements. The two different fiber types have different oxidative abilities. As fat plays an important role in providing energy through oxidation, the generalization of two fiber types as a signal word “muscle” may hinder the understanding of regional lipid accumulation patterns in response to diet. Moreover, these muscles lipids can be further classified into two major pools: (i) EMCL, which resides in adipocytes among the muscle fibers and (ii) IMCL, the intra-myocyte lipid content [4]. It has been suggested that the two lipid pools may have different metabolic roles [5]. Therefore, by separating EMCL and IMCL, a better understanding of “muscle lipids” and its accumulation patterns could be achieved.

MRS can differentiate IMCL and EMCL by their characteristic chemical shifts. Lipids inside the cytosol (IMCL) form small droplets and resonate at 1.3ppm, independent of the relative orientation to the external magnetic field B_0_. Lipids in extracellular adipocytes (EMCL) experience slightly different local magnetism, which shifts their resonance. Their chemical shift is orientation dependent, and is maximal when the muscle fiber is placed parallel to the direction of B_0_; the protons on the methylene groups resonate at 1.5ppm for EMCL (+0.2ppm from IMCL). With adequate resolution, this separation allows quantification of the two different lipid pools [4].

To better understand the role of fiber types in fat accumulation, we examined the lipid deposition in skeletal muscles under short-term high fat diet (HFD) in C57BL/6 mice, a well-accepted model for many aspects of human obesity and metabolic syndrome. Because of the size limitation in skeletal muscles of mice, lumbar muscles and tibialis anterior (TA) were chosen to generate enough MRS signals. It is also technically challenging to find skeletal muscle with a pure fiber type. Therefore, we focused on the effects of fiber composition at the early stage of HFD challenge. Lumbar muscles refer to muscles around the lower spine, which consist of type I fiber predominant muscles including multifidus muscle, interspinales muscle and rotatores muscle [8]. TA muscles are located on the lateral side of the tibia in the leg, and >95% of muscle fibers of TA are type II [9]. In this work, we demonstrate the first MRS application in IMCL/EMCL quantification to mouse spinal lumbar muscles, and present novel comparisons to TA muscles. We hypothesized that muscle fiber composition may affect lipid accumulation patterns in response to diet.

## Material and methods

Healthy male C57BL/6 mice (n = 12) entered the study at 4-weeks old. Mice were randomly divided into two groups, one (n = 8) treated with 60 kcal% HFD for 6 weeks, and the rest (n = 4) remained on normal control diet (NCD) for the same amount of time. The purpose of NCD group is to demonstrate the effectiveness of HFD, and served as controls to clarify that lipid accumulation was induced by diet. In this work, we did not intend to explore the original lipid distribution pattern in the NCD group. At the end of dietary treatment, MRS was performed in mouse liver, TA and lumbar muscles in accordance with guidelines approved by the Institutional Animal Care and Use Committee of Boston University. Then mice were sacrificed, and the weights of their whole body, liver, perigonadal fat, TA and soleus muscles were recorded.

### MRS experiment

MRS experiments were performed with a Bruker 11.7 T Avance spectrometer (Billerica, MA). The mice were anesthetized with 0.5–2% isoflurane and carefully stabilized to achieve the parallel alignment of investigated muscles to the B_0_ field.

**Fig 1:**
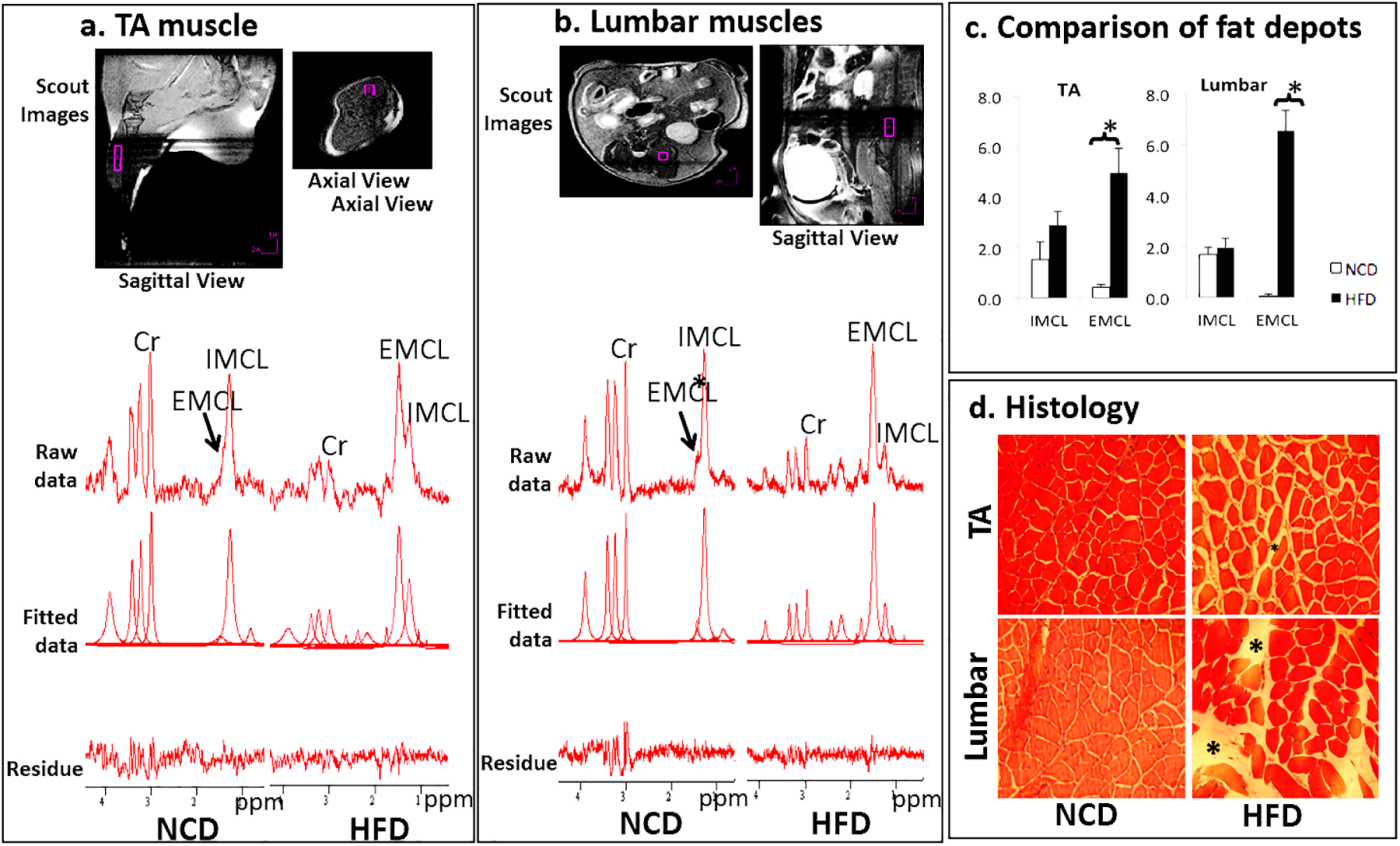
The comparison of tibialis anterior (TA) and lumbar muscles. Representative spectra of TA (a) and lumbar (b) muscles are shown. In both 1a and 1b, scout images (top row) indicate the location of voxels (purple boxes) in both the sagittal and axial view of leg or spinal muscles. Representative spectra (2^nd^ row, raw data) were presented for mice in both normal-control-diet (NCD, left) and high-fat-diet (HFD, right) groups; Raw spectra were analyzed in j-MRUI software to obtain individual fitted component (3^rd^ row, fitted data). The differences between raw data and fitted data in the 4^th^ row (residue); Cr, indicates creatine peak (3.02ppm), and used as reference to measure intramyocellular lipid (IMCL, (1.3ppm) and extramyocellular lipid (EMCL,1.5ppm). (c) The comparison of fat accumulation with and without HFD* Indicates p<0.05. The amount of IMCL/EMCL were recorded as a ratio to Cr-peak, hence it does not associate with an actual unit (y-axis). Representative histology (d) verifies the increases of EMCLs in both TA and lumbar muscles in HFD group. The pale spaces (indicated by “*”) between red stained cell marks the presences of EMCLs, whereas IMCL is not sensitive to the histological staining.

Scout images in the top row (Fig 1A, Fig 1B) were acquired using RARE sequence with: repetition time (TR) = 2500ms, echo time (TE) = 6.5ms, rare factor = 8, slice thickness = 0.5mm. In TA and lumbar muscles: matrix = 192x192, in-plane resolution = 0.156x0.156mm^2^, and in the liver: matrix = 128x128, in-plane resolution = 0.234x0.234mm^2^. In the top row of Fig 1, the axial and sagittal views of the leg (Fig 1A) and the abdominal region (Fig 1B) are used for the geometric planning of MRS acquisition. MRS acquisition voxel indicated by the purple boxes in the top row (Fig 1A, Fig 1B) was carefully placed in the target regions to avoid signal contamination from large fat depots or major vessels. A local shimming was performed before data acquisition. Spectroscopy data was acquired using the PRESS sequence: TR = 2500ms; TE = 8.671ms; bandwidth = 5kHz; sample number = 2048. The voxel sizes are 0.8x0.8x3.5mm^3^, 1.2x1.2x3mm^3^, and 2.5x2.5x2.5mm^3^ for TA, lumbar muscles and liver respectively. For muscles, the water signal was suppressed using VAPOR and 1024 average was used; whereas for liver the average was 128 and without water suppression.

Spectroscopic data was processed using j-MRUI [10]. In muscles, the spectra in the 2^nd^ row raw data (Fig 1A, Fig 1B) were filtered using the HLSVD algorithm to remove residual water signal. The individual spectral components in the 3^rd^ row fitted data (Fig 1A, Fig 1B) were computed through AMARES method [11]. EMCL/IMCL concentrations were expressed relatively as methylene protons to the creatine peak (3.02ppm)[12] for cross sample comparison. Therefore, the amount of EMCL/IMCL reported as unitless. In liver, ICL fat was expressed as protons in–CH2 relative to the water proton peak at 4.7ppm. The ratio of extra/intra-myocellular fat (EIR) was calculated by EMCL divided by corresponding IMCL in the same voxel.

### Histology

Samples of TA/lumbar muscles from each mouse were fixed in a 10% formaldehyde for 3 hours, and stored in 70% ethanol at 4°C until further processing. Tissues were then processed and embedded in paraffin. Five micrometer-thick tissue sections were stained with hematoxylin/eosin. Immunohistochemistry were performed as previously described using Anti-Fast Myosin Skeletal Heavy chain antibody (abcom91506) and Anti-Slow Skeletal Myosin Heavy chain antibody (abcom11083)[13]. Individual positive fiber type in the entire section was counted and quantified with ImageJ[14]. Triglyceride in muscle was biochemically determined using kit assays (Sigma).

### Statistical analysis

Data are presented as mean±SD (standard deviation). Student T-test was performed to compare between HFD and NCD groups. Pearson correlation was used to determine the relationship between parameters. The criteria for statistical significance is p<0.05.

## Results

All 12 mice were successfully examined by MRS. However, one data point from the EMCL in lumbar muscles was excluded due to outlier analysis. Representative spectra of TA/lumbar muscles were shown in Fig 1A and 1B. In all spectra, the IMCL peaks were determined by the methylene proton peak at 1.3ppm, and EMCL at 1.5ppm. In the liver, the ICL peak was determined by a methylene proton peak at 1.3ppm.

HFD significantly increased body and liver weight, as well as perigonadal fat (all p<0.001). TA muscle of HFD (40.7±2.6 g) was lighter than NCD mice (55.3±2.4g, p<0.01) whereas soleus muscle weight was preserved (p = 0.36). Characteristics of the mice and the average amount of fat depots are summarized in Table 1. Among all three intracellular fat depots, only ICL (liver) showed a significant increase with the HFD compared to the NCD (p = 0.01). The amount of IMCL in TA increased ~2 fold but was not statistically different (p>0.05) between the two groups (Fig 1C). In lumbar muscles (Fig 1C), the IMCLs were similar with (2.0±0.4) or without (1.7±0.3) HFD. The EMCLs were significantly elevated with HFD in both types of muscles. In TA, the average EMCL in NCD group was 0.4±0.1, and in the HFD group increased ~12 fold to 5.0±1.0 with p<0.01 (Fig 1C, Fig 1D). In lumbar muscles, the EMCL increased even more ~66 fold with p<0.001 (Fig 1C, Fig 1D). The total fat (IMCL+EMCL) showed significant increases (all p<0.01) comparing NCD and HCD groups in both muscle types. As for EIR, there was no significant difference between NCD and HFD for TA muscles, whereas in lumbar muscles, EIR significant increased after HFD (p<0.01). Representative histological data confirmed that EMCL accumulated to a greater extent in lumbar muscles than in TA muscles (Fig 1D).

**Table 1:** The characteristics of NCD and HCD mice.

	NCD	HFD	p
Body weight (g)	21.1±0.7	38.6±1.8	<0.001
Tissue weight (mg)			
TA muscle	55.3±2.4	40.7±2.6	<0.01
Soleus muscle	9.8±0.7	8.9±0.6	0.36
Liver	1458±24.1	1782±32.1	<0.001
Perigonadal fat	479±57.4	2520±176	<0.001
MRS*			
TA muscle			
IMCL	1.5±0.7	2.9±0.5	0.16
EMCL	0.4±0.1	5.0±1.0	<0.01
Total	2.0±1.2	7.9±2.8	<0.01
EIR	0.7±0.0	2.3±1.9	0.14
Lumbar muscles			
IMCL	1.7±0.3	2.0±0.4	0.64
EMCL	0.1±0.1	6.6±0.8	<0.001
Total	1.7±0.9	8.5±3.0	<0.01
EIR	0.0±0.1	4.2±2.2	<0.01
Liver			
ICL	0.0±0.0	0.1±0.0	0.01
Triglyceride (μg/mg)			
TA muscle	16.1±0.6	34.9±1.7	<0.001
Lumbar muscles	39.9±10.3	118.2±20.3	<0.05

*The amount of fat was expressed as the percentage of peak value ratio of lipid to reference peak by 1H MRS. The reference peak for liver fat was the water signal at 4.7ppm, and for muscle fat was the creatine signal at 3.02ppm.

**Fig 2:**
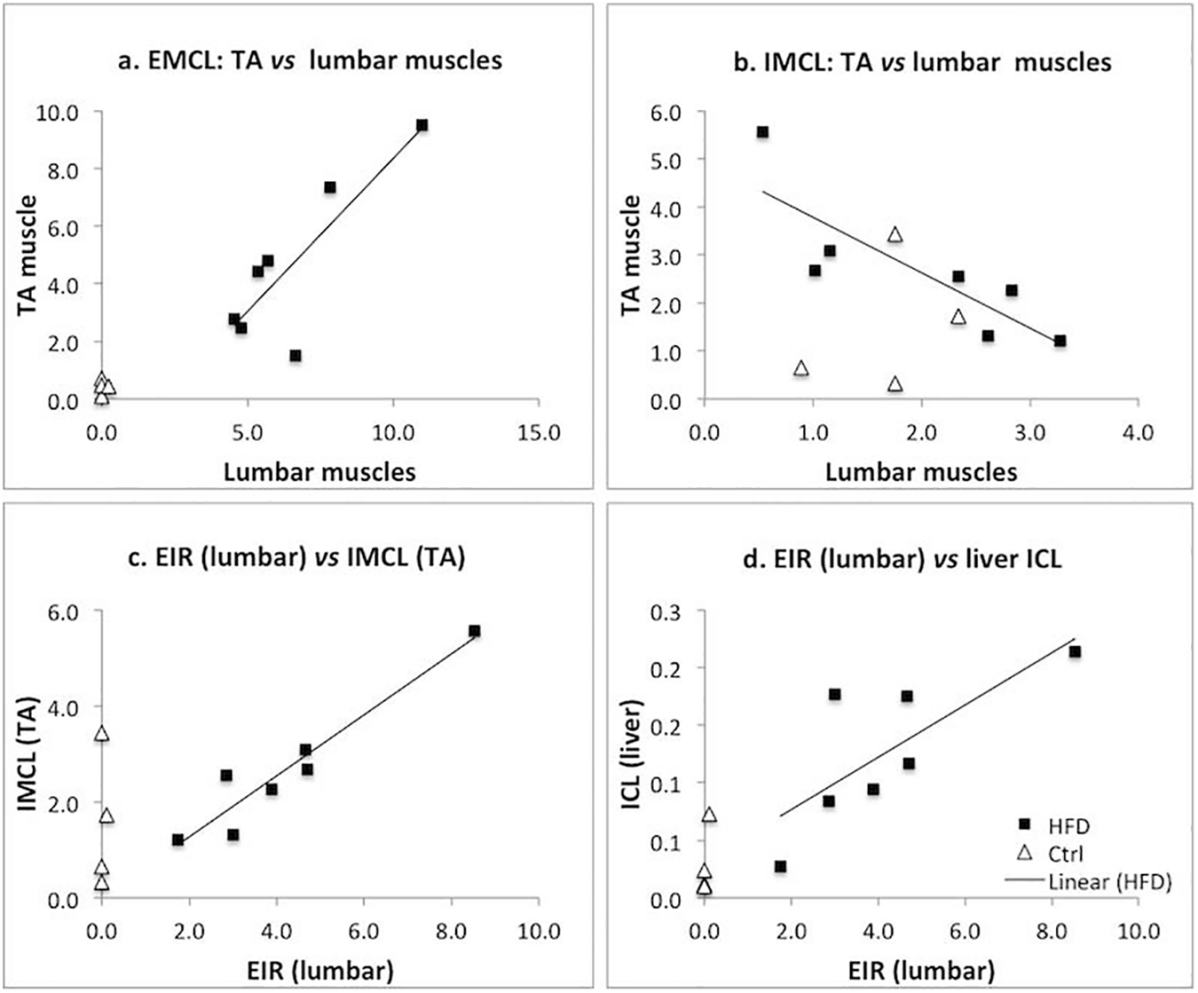
Correlations between anatomically distant fat depots. (a) EMCL in HFD mice are highly correlated (r = 0.84, p = 0.01) between TA and lumbar muscles (squares). (b) The smaller depot of IMCL is negatively correlated between muscle types in HFD (r = -0.84, p = 0.01). (c) TA IMCL is correlated with the ratio of EMCL to IMCL (EIR) (r = 0.96, p<0.001, and (d) liver ICL (r = 0.77, p = 0.03) fat is correlated with lumbar EIR. Data for control mice are shown in triangles.

MRS data also made it possible to assess relationships between different fat depots. Anatomically adjacent IMCL and EMCL were not correlated with each other (all p>0.05). However, the results for distant fat depots were intriguing, especially in the HFD group. EMCLs in both muscles were highly correlated with r = 0.84 and p = 0.01 (Fig 2A), but we found an unexpected negative correlation with r = -0.84 and p = 0.01 (Fig 2B) of IMCLs between TA and lumbar muscles. Another interesting finding is that EIR of lumbar muscles showed a stronger correlation to distant intracellular fat (TA IMCL: r = 0.96, p<0.001; liver ICL: r = 0.77, p = 0.03) (Fig 2C, Fig 2D), as compared to that of EIR in TA muscle (lumbar IMCL: r = 0.75, p = 0.04; liver: p = 0.61).

**Fig 3:**
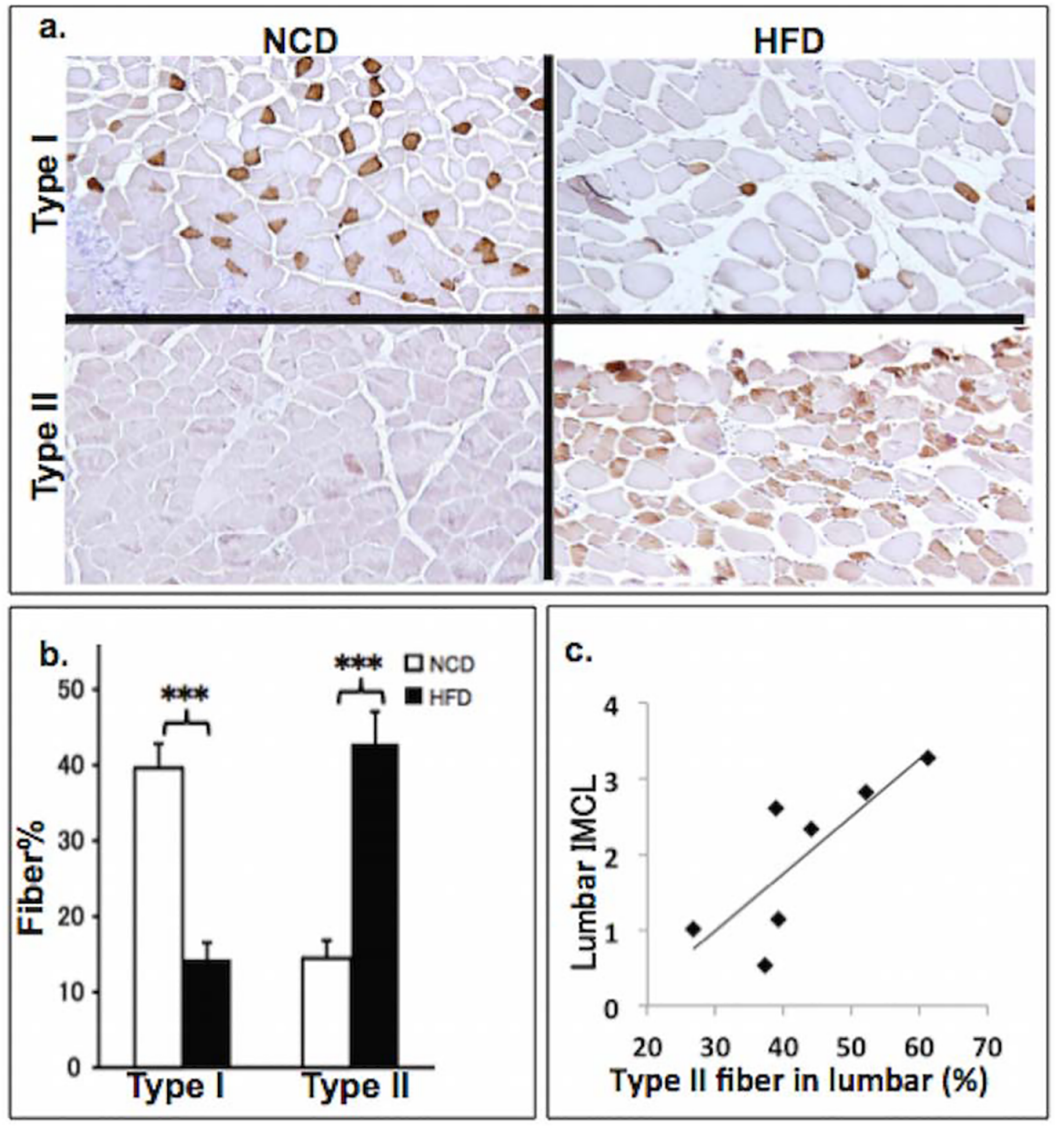
Fiber type analysis of lumbar muscles. (**a**) Immunohistochemistry for type I and type II fiber in lumbar muscles. (**b**) Comparison of fiber type percentage in lumbar muscles between normal-control-diet (NCD) and high-fat-diet (HFD) mice. Type I fiber significantly decreased (p<0.001) and type II fiber significantly increased (p<0.001) in HFD mice (**C**) Correlation between intramyocellular lipid (IMCL) and percentage of type II fiber in lumbar muscle of HFD mice. IMCL positively correlates to percentage of type II fiber in HFD mice (r = 0.80, p = 0.03).

Skeletal muscle may shift fiber composition in response to physiological changes such as HFD and exercise training [15–17]. We discovered that fiber shifting may relate to original fiber composition. In lumbar muscles, HFD significantly decreased type I fiber while it increased type II fiber (Fig 3A, Fig 3B). In TA muscle, there was no significant fiber type shifting (all p>0.05). Interestingly, the increased type II fiber percentage significantly correlated to IMCL in lumbar muscle of HFD mice with r = 0.80 and p = 0.03 (Fig 3C).

## Discussion

Recent studies have shown that lipid distribution plays an important role in health consequences [18–20]. In this study, we focused on the impact of fiber compositions on the early development of muscular adiposity. In the same muscle, EMCL and IMCL are anatomically close to each other, yet these distinct pools showed different initial responses to HFD. Similar to previous studies with mice [21,22], our short-term HFD treatment increased EMCL in both TA and lumbar muscles; yet surprisingly had limited effects on IMCL, which suggests that fat tends to accumulate first into extramyocellular adipocytes. The strong correlation between EMCLs in distant muscles suggests that extra-cellular adipocytes share similar properties and work as lipid storage buffers independent from muscle fiber types and location in the early phase of metabolic disorder.

It is interesting, that IMCL is initially unaffected by HFD. Short-term HFD increases the gene expression of proteins involved in the β-oxidation pathway [23]. Hence, it is possible that skeletal muscles may resist to lipid stress through lipid consumption, yet this ability may vary depending on fiber compositions. We discovered that IMCL in lumbar muscles remained at a similar level after the short HFD while TA showed ~2 fold elevation. Compared to TA muscle, the majority of lumbar muscles, including multifidus muscle, interspinales muscle and rotatores muscle, are dominated by type I fibers [8], which contain large quantities of mitochondria, and utilize fatty acids through β -oxidation as a major energy source. Higher β-oxidation capacity of lumbar muscles may contribute to the tolerance for IMCL accumulation in the early phase of obesity. However, it is also evident that lumbar muscles start shifting towards less oxidative fiber after HFD. Therefore its tolerance to lipid flux may be mitigated later. In fact, we observed that its level of fiber shifting significantly relates to IMCL. However, further work is needed to clarify whether fiber shifting is the result or precursor of impaired β-oxidation.

Ideally, skeletal muscles in anatomic proximity, for example, soleus (type I preference) vs TA (type II preference) muscles, should also be investigated to mitigate the location influences of fat accumulation. However, highly obese mice are too large to fit into the probe and hence, the size of mice limited our choices of muscles. Another limitation of our study is that MRS was acquired in only one voxel for each targeted muscle per mouse. Hence, with mice, a selection bias may exist and future studies in large animals or human will be valuable.

In conclusion, our study demonstrated fiber composition might affect skeletal muscles responses to excess dietary lipid at early stage. At this stage, oxidative type I muscles showed more resistance to lipid stress, yet were more susceptible to fiber shifting. This may suggest muscle might compensate the systemic metabolic overload with regulation of fat depot and fiber type shift.
